# Effect of strikes by health workers on mortality between 2010 and 2016 in Kilifi, Kenya: a population-based cohort analysis

**DOI:** 10.1016/S2214-109X(19)30188-3

**Published:** 2019-05-22

**Authors:** Gerald Ong'ayo, Michael Ooko, Ruth Wang'ondu, Christian Bottomley, Amek Nyaguara, Benjamin K Tsofa, Thomas N Williams, Philip Bejon, J Anthony G Scott, Anthony O Etyang

**Affiliations:** a Department of Epidemiology and Demography, KEMRI-Wellcome Trust Research Programme, Kilifi, Kenya; b Yale University, New Haven, CT, USA; c Department of Infectious Disease Epidemiology, London School of Hygiene & Tropical Medicine, London, UK; d Imperial College, London, UK; e Nuffield Department of Medicine, University of Oxford, Oxford, UK

## Abstract

**Background:**

Health workers' strikes are a global occurrence. Kenya has had several strikes by health workers in recent years but their effect on mortality is unknown. We assessed the effect on mortality of six strikes by health workers that occurred from 2010 to 2016 in Kilifi, Kenya.

**Methods:**

Using daily mortality data obtained from the Kilifi Health and Demographic Surveillance System, we fitted a negative binomial regression model to estimate the change in mortality during strike periods and in the 2 weeks immediately after strikes. We did subgroup analyses by age, cause of death, and strike week.

**Findings:**

Between Jan 1, 2010, and Nov 30, 2016, we recorded 1 829 929 person-years of observation, 6396 deaths, and 128 strike days (median duration of strikes, 18·5 days [range 9–42]). In the primary analysis, no change in all-cause mortality was noted during strike periods (adjusted rate ratio [RR] 0·93, 95% CI 0·81–1·08; p=0·34). Weak evidence was recorded of variation in mortality rates by age group, with an apparent decrease among infants aged 1–11 months (adjusted RR 0·58, 95% CI 0·33–1·03; p=0·064) and an increase among children aged 12–59 months (1·75, 1·11–2·76; p=0·016). No change was noted in mortality rates in post-strike periods and for any category of cause of death.

**Interpretation:**

The brief strikes by health workers during the period 2010–16 were not associated with obvious changes in overall mortality in Kilifi. The combined effects of private (and some public) health care during strike periods, a high proportion of out-of-hospital deaths, and a low number of events might have led us to underestimate the effect.

**Funding:**

Wellcome Trust and MRC Tropical Epidemiology Group.

## Introduction

Strikes by health workers are a global occurrence.^1^ Strikes are caused by many factors, such as suboptimum remuneration,^2^ unfavourable working hours,^3^ high malpractice insurance premiums,^4^ poor working conditions,^5^, ^6^, ^7^ and harassment by patients' relatives.^8^ There has been much debate about whether it is ethical to withdraw services deemed essential for survival and on the potential detrimental effect of such strikes on mortality.^9^, ^10^

Many strikes by health workers have been reported in sub-Saharan Africa, with one study estimating that there were 620 strikes between 1996 and 2015, of which about a fifth were nationwide (Friedman WH, University of Houston, Houston, TX, USA, and Keats A, Wesleyan University, Middletown, CT, USA, personal communication). Assuming an equal distribution of strikes over time, this number would translate to each country having a strike about once every 18 months. Most published work on the effects of health workers' strikes on mortality are from high-income settings, with three reports identified from sub-Saharan Africa.^6^, ^11^, ^12^ The Kenyan health sector has had more than 40 regional and national strikes by health workers since 1998 (Friedman WH and Keats A, personal communication), with the most recent entailing a 100-day doctors' strike followed by a 5-month nurses' strike, during which time services in all public hospitals in the country were disrupted.^11^, ^13^ Although it is evident that strikes by health workers reduce provision of and access to medical services,^6^, ^12^, ^14^, ^15^ the reported effect on mortality has been varied. Some studies have reported no change in mortality,^15^, ^16^, ^17^ others an increase in deaths,^6^, ^11^, ^18^ yet other studies have shown an unexpected decrease in mortality.^4^, ^19^ Findings of no change in mortality have been attributed largely to continued access to emergency services,^1^, ^20^ partial involvement of striking staff, and continued service provision by other cadres of medical staff.^14^, ^15^, ^17^ Reduced mortality has been ascribed to withdrawal of elective surgical procedures and continued provision of emergency care,^1^, ^19^, ^21^ whereas increased mortality has been attributed to poorer quality of care^6^, ^18^ and reduced access to emergency services.^11^

Most strikes by health workers in developing countries, including Kenya, leave no residual provision for emergency services in public health-care facilities. The added burden of poor socioeconomic status and deficient infrastructure means that such service disruptions in low-income countries can have a more severe effect than in high-income settings.^9^ Therefore, mortality rates—particularly those among patient populations in most need of emergency care—might be increased disproportionately by health workers' strikes in these countries. However, in view of the varied outcomes of studies assessing the effect of health workers' strikes in other parts of the world, it is difficult to predict the effect in sub-Saharan Africa. Deficiencies in civil registration processes in regions in sub-Saharan Africa also restrict the ability to measure the effect of strikes on the population. However, within Health and Demographic Surveillance Systems, which monitor community-based mortality rates, the effect of strikes on the population can be assessed, and this research might provide insights to guide public health strategies to mitigate the risks of health workers' strikes.^16^ We analysed the effect of six nationwide health workers' strikes that occurred between 2010 and 2016 on mortality in Kilifi county in coastal Kenya, using data from the Kilifi Health and Demographic Surveillance System (KHDSS).

**Research in context**

**Evidence before this study**

We searched PubMed with no date restriction for studies on health workers' strikes published in English, using the terms “doctors” or “nurses” or “health workers” and “strikes” or “industrial action”. We also included relevant articles referenced in the retrieved publications. Although strikes by health workers have occurred across the world, most published work on their effects is from high-income settings. Strikes in this setting are characterised by continued service provision (in the form of emergency care as a minimum), with other strikes having either near-normal health-care provision because of partial involvement of striking staff or redistribution of duties to non-striking staff. These strikes by health workers were largely associated with no change or a decrease in mortality, mostly attributable to fewer deaths from elective surgical procedures. Some studies, however, reported an increase in mortality during the strikes they analysed. Only a few studies of the effect of strikes were in low-income settings, with two reports of increased mortality and one of fewer deaths. These were hospital-based studies and did not account for deaths that occurred outside the respective hospitals.

**Added value of this study**

Using demographic surveillance data obtained over 7 years, we analysed the effect of six strikes by health workers that occurred within that period on mortality in Kilifi, Kenya. The surveillance data captured all deaths that happened, both in and out of hospital. The causes of death were derived from contemporaneously obtained verbal autopsy data. Our findings suggest that, overall, mortality did not change during the strikes, but there was a possibility of increased deaths in children aged 1–5 years.

**Implications of all the available evidence**

Strikes of relatively short duration are less likely to have discernible effects on mortality. Continued service delivery—even at an emergency level—is likely to avert adverse effects of health workers' strikes.

## Methods

### Study setting and data collection

We obtained data for our study from the KHDSS. This health database covers an area of 891 km^2^ comprising both rural and semiurban regions in Kilifi county in coastal Kenya, which has a population of approximately 300 000 people.^22^ Information on pregnancies, births, deaths, and migrations within the KHDSS is updated every 4 months, and cause of death data are obtained using verbal autopsies.^23^ The crude death rate within the KHDSS for the period 2006–10 was 5·85 deaths per 1000 person-years of observation,^22^ and the prevalence of HIV in Kilifi county in 2015 was 4·4%.^24^

The main referral facility within the KHDSS is the Kilifi County Hospital (KCH), which is a level 4 government-run facility^25^ that provides both inpatient and outpatient services. Additional facilities within the KHDSS comprise three government-run health centres, 16 dispensaries, and approximately 42 private health facilities, of which only about 10% offer inpatient services. The proportion of babies born at home within the KHDSS decreased from 53% in 2012 to 30% in 2016. The proportion of babies in the KHDSS born at a health facility increased from 44% in 2012 to 68% in 2016.

At KCH, inpatient services are provided for adults in the adult and maternity wards and for children in the general paediatric ward and in the high dependency unit (HDU). The HDU is run by the KEMRI-Wellcome Trust Research Programme. On average, 20% of paediatric patients are admitted to the HDU and 80% to the general paediatric ward. The general paediatric ward has a 70-bed capacity and is staffed on average with two nurses, five clinical officer interns, two medical officer interns, and one consultant paediatrician. The HDU has six beds, six cots, and four incubators and is staffed on average with three nurses, three clinical officer interns, one medical officer intern, and two consultant paediatricians. Approximately 4% of all deaths recorded in the KHDSS and 38% of all paediatric inpatient deaths occur in the HDU.

During strikes by health workers in the period 2010–16, service delivery was disrupted by the striking primary staff cadre (eg, doctors), which led to hampered service delivery by other non-striking staff cadres (eg, nurses). All non-striking staff were expected to be present at their respective facilities. During strike periods, the HDU at KCH remained operational but limited paediatric inpatient services were provided. Admissions to this unit were restricted to the most critical cases because of the limited capacity, although there was a provision for multiple patients to share a bed. Staff in the HDU provided services only within this unit and were not redistributed to other hospital departments to offer services such as high-risk deliveries or caesarean sections. No arrangements were made for hiring replacement staff at KCH during strike periods.

Mortality data within the KHDSS were obtained with approval from the Kenya Medical Research Institute Scientific Ethics Review Unit (SSC 1348).

### Procedures

We ascertained the dates of strikes by doctors, nurses, or both by searching through digital archives of Kenyan newspapers. We confirmed these dates by checking admission data from KCH. A strike was defined by a national announcement of cessation of service provision by doctors, nurses, or both in government hospitals across the country and a concomitant decline in admissions at KCH.

We obtained dates of death from an existing electronic database of admissions to KCH (maintained by KEMRI-Wellcome Trust Research Programme), from people living in the same or a neighbouring homestead who knew the deceased, or by both these ways. In the primary analysis, we excluded deaths for which the exact date on which the death occurred was not known.

The routine verbal autopsy and cause of death allocation process in the KHDSS has been previously described.^23^ Briefly, people with information on the deceased were interviewed using standard WHO verbal autopsy questionnaires. From their responses, causes of death were assigned using the InterVA-4 computer-based probabilistic model.^23^ We categorised causes of death assigned by verbal autopsy into six broad groups—maternal, medical, surgical, medical or surgical, trauma-associated, and other causes. We defined childbearing age as 15–49 years old.

### Statistical analysis

The period of analysis was from Jan 1, 2010, to Nov 30, 2016. We did not include December, 2016, in our analysis because another strike by health workers started at the beginning of this month and continued into 2017, covering a period for which KHDSS data were not available at the time of analysis. The mid-month population in the KHDSS was used to estimate person-days of observation.

We combined all strike periods to form a strike days category (referred to as the strike period) and all non-strike periods to form a non-strike days category (referred to as the non-strike period). We compared mortality during the strike period with mortality during the non-strike period using negative binomial regression to account for overdispersion. We used Newey West SEs to adjust for autocorrelation, allowing a lag of up to 7 days.^26^ We calculated the maximum non-zero lag using the formula 4(*n*/100)^2/9^, for which *n* refers to the length of the time series.^26^ We adjusted for categorical variables that we had identified a priori as possible confounders—namely, year and month (adjusting for long-term mortality trends and seasonality, respectively), day of the week, and public holiday. Prespecified subgroup analyses included comparisons of mortality during the strike and non-strike periods by age group, cause of death, and strike week.

We investigated possible delayed effects of strikes on mortality by comparing mortality in the first and second weeks immediately after a strike with mortality in the non-strike period. We checked whether inclusion of interaction terms for age group and strike week improved the regression model using a multiparameter Wald test. The reference category in the strike week analysis was the non-strike period. Finally, we did two sensitivity analyses to examine the effect of excluding deaths with missing dates. In the first sensitivity analysis, we included all these deaths but assigned the date of death as the 15th of the month in which the death was reported to have occurred, and we included an indicator variable for day 15 in the model. In the second analysis, we ran the regression analysis on 100 imputed datasets, in which the date of death was randomly assigned to any day within the month in which it occurred.

We did statistical analyses with Stata, version 15.1.

### Role of the funding source

The funder had no role in study design, data collection, data analysis, data interpretation, or writing of the report. The corresponding author had full access to all data in the study and had final responsibility for the decision to submit for publication.

## Results

Between Jan 1, 2010, and Nov 30, 2016, six strike periods were identified, ranging in length from 9 days (Dec 5–13, 2011) to 42 days (Dec 3, 2012, to Jan 13, 2013), with a median strike duration of 18·5 days (table 1). A 1-day county-level strike was noted during this period, but this strike was not included in the analysis. The analysis covered a period of 2525 days, of which 128 (5%) were designated as strike days.

**Table 1:** Characteristics of nationwide strikes by health workers that occurred between January, 2010, and November, 2016, and effects within the KHDSS

	**Duration (days)**	**Strike participants**	**Deaths (n)**	**Person-days**	**Crude mortality rate (per 100 000 person-days of observation)**
Dec 5 to 13, 2011	9	Doctors	53	5 210 802	1·02
March 1 to 15, 2012	15	Nurses	102	8 146 404	1·25
Sept 13 to Oct 4, 2012	22	Doctors	120	12 377 464	0·97
Dec 3, 2012, to Jan 13, 2013	42	Nurses	231	24 314 926	0·95
Jan 16 to Feb 11, 2013	26	Nurses	104	16 046 767	0·65
Dec 10 to 23, 2013	14	Doctors and nurses	57	7 838 181	0·73

KHDSS=Kilifi Health and Demographic Surveillance System.

At the mid-point of the analysis period (June 15, 2013), the KHDSS population comprised 274 971 people, with 48 456 (18%) aged younger than 5 years, 132 959 (48%) aged younger than 15 years, and 28 063 (10%) aged 50 years and older. During the analysis period, 1 829 929 person-years (668 381 702 person-days) of observation were recorded. The strike period comprised 33 943 343 person-days of observation and the non-strike period consisted of 634 438 359 person-days of observation. Inpatient admission rates at KCH were 3·17 per 100 000 person-days during the non-strike period and 1·04 per 100 000 person-days during the strike period.

Between Jan 1, 2010, and Nov 30, 2016, 9159 deaths were recorded among KHDSS residents. Of these, the date of death was uncertain in 2763 (30%) cases, which were excluded from the primary analysis (figure). The age distribution of deaths excluded from the analysis did not differ from those that were included (data not shown). Therefore, 6396 (70%) deaths were included in the analysis, of which 1414 (17%) occurred in children aged younger than 5 years, 345 (5%) in children aged 5–14 years, 1467 (25%) in people aged 15–49 years, and 3170 (53%) in adults aged 50 years or older. 6100 (95%) of 6396 deaths occurred during the non-strike period, of which 4713 (77%) were reported to have occurred outside the hospital.

**Figure:**
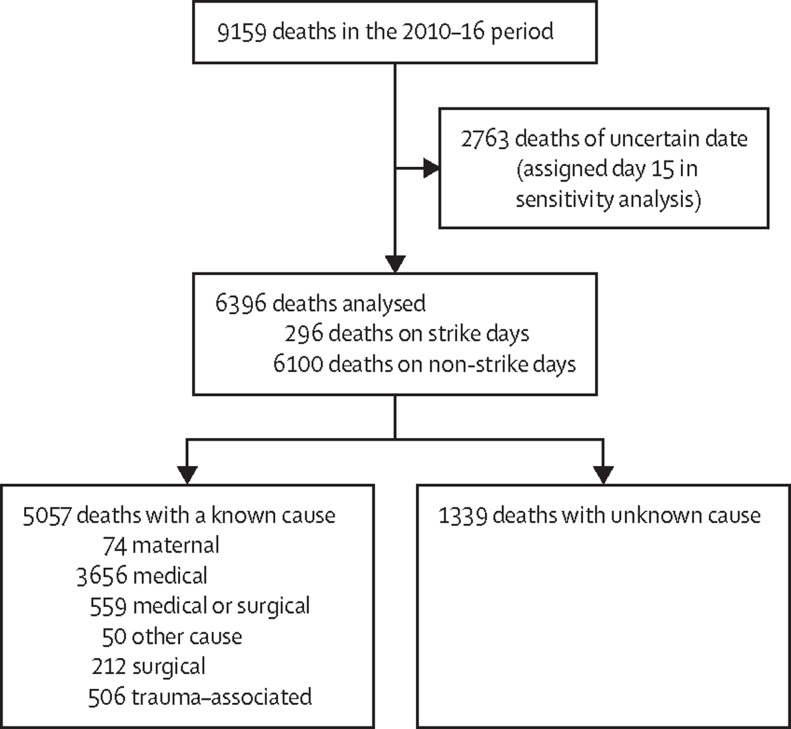
Deaths included in the overall analysis and causes of death

The crude death rate in the strike period was 0·87 per 100 000 person-days of observation and in the non-strike period it was 0·96 per 100 000 person-days of observation. In regression analyses, no change was noted in mortality during the strike period compared with the non-strike period (adjusted rate ratio [RR] 0·93, 95% CI 0·81–1·08; p=0·34; table 2).

**Table 2:** Effect of strikes by health workers on mortality in the KHDSS, both overall and by age categories, between January, 2010, and November, 2016

	**Deaths (n)**	**Person-days**	**Mortality rate (per 100 000 person-days of observation)**	Rate ratio (95% CI)*	**p value**
**Overall**					
Non-strike days	6100	634 438 359	0·96	1·00	..
Strike days	296	33 943 343	0·87	0·93 (0·81–1·08)	0·34
**Age <1 month**					
Non-strike days	663	4 179 963	15·86	1·00	..
Strike days	32	208 427	15·35	0·97 (0·69–1·37)	0·88
**Age 1–11 months**					
Non-strike days	320	19 619 646	1·63	1·00	..
Strike days	11	1 059 244	1·04	0·58 (0·33–1·03)	0·064
**Age 12–59 months**					
Non-strike days	356	87 050 849	0·41	1·00	..
Strike days	32	4 780 187	0·67	1·75 (1·11–2·76)	0·016
**Age 5–14 years**					
Non-strike days	330	194 417 465	0·17	1·00	..
Strike days	15	10 399 895	0·14	0·90 (0·46–1·76)	0·75
**Age 15–49 years**					
Non-strike days	1391	264 336 791	0·53	1·00	..
Strike days	76	14 061 678	0·54	1·01 (0·78–1·30)	0·96
**Age ≥50 years**					
Non-strike days	3040	64 833 645	4·69	1·00	..
Strike days	130	3 433 912	3·79	0·84 (0·68–1·04)	0·10

KHDSS=Kilifi Health and Demographic Surveillance System.

* Rate ratio adjusted for trend, seasonality, day of the week, and public holiday.

A 75% increase in mortality was noted during the strike period among children aged 12–59 months (adjusted RR 1·75, 95% CI 1·11–2·76; p=0·016). Among infants aged 1–11 months, weak evidence was found for a decrease in all-cause mortality during the strike period (adjusted RR 0·58, 95% CI 0·33–1·03; p=0·064). No change in mortality between the strike period and non-strike period was seen in other age categories (table 2). In a model that included an interaction term between age group and strike period, weak evidence was found that the mortality rate recorded during the strike period in children aged 12–59 months differed significantly from that of the baseline age group (babies <1 month; p=0·051).

Information on cause of death was available for 5057 (79%) deaths that occurred during the analysis period. 3656 (72%) deaths were attributable to medical causes, 559 (11%) to medical or surgical causes, 506 (10%) to trauma-associated causes, and 212 (4%) to surgical causes (appendix pp 1, 2). No change was noted in mortality during the strike period for any of the cause of death categories (table 3).

**Table 3:** Effect of strikes by health workers on mortality by cause of death in the KHDSS between January, 2010, and November, 2016

	**Deaths (n)**	**Mortality rate (per 100 000 person-days of observation)**	Rate ratio (95% CI)*	**p value**
**Maternal cause of death**				
Non-strike days	71	0·05	1·00	..
Strike days	3	0·04	0·70 (0·22–2·20)	0·55
**Medical cause of death**				
Non-strike days	3477	0·55	1·00	..
Strike days	179	0·53	0·93 (0·78–1·10)	0·41
**Medical or surgical cause of death**				
Non-strike days	528	0·08	1·00	..
Strike days	31	0·09	1·15 (0·76–1·73)	0·51
**Other cause of death**				
Non-strike days	47	0·01	1·00	..
Strike days	3	0·01	1·16 (0·34–3·99)	0·81
**Surgical cause of death**				
Non-strike days	207	0·03	1·00	..
Strike days	5	0·01	0·59 (0·24–1·46)	0·26
**Trauma-associated cause of death**				
Non-strike days	470	0·07	1·00	..
Strike days	36	0·11	1·09 (0·75–1·59)	0·64
**Unknown cause of death**				
Non-strike days	1300	0·20	1·00	..
Strike days	39	0·11	0·78 (0·52–1·17)	0·23

KHDSS=Kilifi Health and Demographic Surveillance System.

* Rate ratio adjusted for trend, seasonality, day of the week, and public holiday.

Some differences were noted in mortality by strike weeks compared with the non-strike period (table 4), but these differences were not significant (p_interaction_=0·12).

**Table 4:** Mortality by strike week in KHDSS residents

	**Deaths (n)**	**Mortality rate (per 100 000 person-days of observation)**	Rate ratio (95% CI)*	**p value**
No strike	6100	0·96	1·00	..
Week 1	95	0·88	0·93 (0·74–1·17)	0·53
Week 2	78	0·80	0·83 (0·68–1·02)	0·077
Week 3	50	0·88	0·95 (0·71–1·26)	0·72
Week 4	30	0·79	0·75 (0·57–0·98)	0·033
Weeks 5 and 6	43	1·13	1·36 (1·11–1·67)	0·0030

KHDSS=Kilifi Health and Demographic Surveillance System.

* Rate ratio adjusted for trend, seasonality, day of the week, and public holiday.

No change in mortality was noted in the first week immediately after a strike compared with the non-strike period (adjusted RR 1·12, 95% CI 0·95–1·33; p=0·18). Weak evidence was found for increased mortality in the second week after a strike (adjusted RR 1·15, 95% CI 1·00–1·32; p=0·057). No evidence of interaction between age group and mortality was noted during the first week (p=0·79) and the second week (p=0·083) after a strike.

An effect of weekdays and public holidays on mortality was recorded (appendix p 3), with a reduction in deaths during weekends (adjusted RR 0·87, 95% CI 0·82–0·93; p<0·0001), and weak evidence of an increase in mortality during public holidays (1·14, 0·99–1·30; p=0·067).

Overall mortality rate ratios were similar in the primary analysis excluding deaths of uncertain date (adjusted RR 0·93, 95% CI 0·81–1·08; p=0·34) and in the sensitivity analyses when deaths of uncertain date were assigned to day 15 (adjusted RR 0·94, 95% CI 0·83–1·08; p=0·39) and in the imputation analysis (1·01, 0·86–1·16; p=0·60). Mortality rate ratios were also similar in the age-specific subanalyses (appendix p 4).

## Discussion

In our community-based analysis of the effect of six country-wide strikes by health workers on mortality in Kilifi county in Kenya, we recorded no change in overall mortality during strike periods compared with non-strike periods, and no changes were seen in cause-specific mortality. Weak evidence was found for increased mortality during strike periods among children aged 12–59 months, but this finding could be attributable to chance.

Our findings of no change in overall mortality between strike and non-strike periods have also been noted in several regions after strikes by doctors or nurses, including in Alberta (Canada),^15^ Croatia,^16^ England,^14^ New Zealand,^27^ and Spain.^17^ However, differences between these studies and ours make it difficult to compare results. For example, some analyses were limited by age distribution or focused on selected units in a hospital,^15^, ^17^ whereas others did not report age-stratified results.^14^, ^27^ Furthermore, very few reports of the effects of strikes by health workers have been from low-to-middle income settings, and those that were did not assess mortality at the population level.^5^, ^6^, ^11^, ^12^

Reasons for no change in mortality in settings other than ours include continued provision of emergency services during strikes,^1^, ^16^, ^19^, ^20^ partial involvement of doctors or nurses in strikes,^2^, ^4^, ^14^ stand-in staff replacements during strikes,^2^, ^17^, ^27^ and strikes lasting for too short a duration to have a substantial effect.^20^ Strikes have also been associated with a reduction in exposure to potentially harmful interventions, such as elective surgical procedures,^10^, ^20^, ^21^ and sometimes an overall decrease in mortality was recorded during strikes.^1^, ^19^, ^20^

During the nationwide strikes in Kenya, although service delivery (including emergency services such as caesarean sections) was affected in public hospitals including KCH, health-care provision was continued in private facilities and at hospitals run by faith-based organisations. In Kenya, approximately 38% of health facilities are managed by the private sector, 10% by faith-based organisations, and 3% by non-governmental organisations, with the remaining 49% run by the government.^28^ Similarly, in Kilifi county, approximately 48% of health facilities are managed by the private sector and 5% by faith-based organisations.^28^ Reports from Kenyan media indicated increased numbers of patients in private facilities during the strikes by health workers.^29^ KCH also had continued provision of care at the paediatric HDU for critically ill patients throughout the strikes. This continued service delivery would, in part, mitigate the effect of the strikes by health workers.

Although we did not see a reduction in surgery-related mortality between strike and non-strike periods, patients within the KHDSS were exposed to other risks related to health-care provision. For paediatric inpatients, hospital-acquired infections confer more than twice the mortality risk when compared with community-acquired infections,^30^ and intravenous fluid bolus treatment for severely ill children is associated with increased mortality.^31^ Reduced exposure to some of these potentially harmful interventions would, in part, mitigate the mortality risk during the strikes by health workers and balance out the effect of the reduced access to health-care services.

Another explanation for recording no change in mortality between strike and non-strike periods could be related to suboptimum quality of services being provided during regular operation of health facilities. If health-care provision is compromised under normal circumstances, the effect of its cessation would be marginal. One metric of service provision is the World Bank Service Delivery Indicators Survey, which gathered data from 294 health facilities and 1859 health providers across Kenya in 2012.^32^ From the report, none of the public facilities had all the essential drugs for children and women (average availability was 67%) and 28% of facilities did not have the basic minimum equipment expected, with problems being worse in rural facilities.^32^

A further reason for no change in mortality between strike and non-strike periods could be that many deaths in low-income settings occur outside hospital. In our analysis, 77% of deaths in the non-strike period were outside hospital. Therefore, a strike by health workers in curative health services might have less effect if a large proportion of the population are not attending hospital for treatment, and the effect of a strike on preventive public health services is likely to be delayed and less obvious if the strike is of short duration. Furthermore, even after life-saving treatment has been administered during an inpatient stay, post-discharge mortality remains high, implying that the effect of hospital could be to delay—but not prevent—death in some cases.^33^

The relatively short duration of strikes by health workers could also have led to the failure to record an effect on mortality, as suggested previously by Cunningham and colleagues.^20^ There were relatively few deaths in these short strike periods, after excluding deaths when timing was uncertain. Thus, our analysis had low power to detect modest effects. The role of Kenyan hospitals in child survival is complex and difficult to disentangle from preventive care.^34^ Examining the effects of longer strikes—eg, those that occurred in 2017, which had a greater effect on admission rates than did the strikes reported here^13^—might be able to confirm this hypothesis.

Limitations of our study include the exclusion of a proportion of deaths because of uncertainty surrounding the actual date of death, which inadvertently led to underestimation of mortality rates. However, exclusion of uncertain dates helped avoid potential misclassification of deaths and improved the accuracy of our estimates. The sensitivity analysis, which included all deaths, yielded similar results to the primary analysis. Another limitation is that our study was not adequately powered to investigate the effect of specific causes of death within age groups, which would have helped us to better understand the possible reasons for the apparent increased mortality in children aged 12–59 months.

Strengths of our study include use of population-level data, which overcome limitations inherent in using hospital-based data that do not account for deaths reported outside the hospital. We also incorporated data from multiple strikes occurring at different timepoints, thereby increasing the power of the study to detect a change in addition to mitigating the effects of seasonality of mortality rates. Moreover, the regression model we used adjusted for possible confounders including weekends, public holidays, mortality trends, and seasonality, further refining our estimates.

From our analysis, we conclude that the short strikes by health workers in 2010–16 had no discernible effect on overall mortality in Kilifi, Kenya. The combined effects of private (and some public) health care during strike periods, the high proportion of out-of-hospital deaths and post-discharge deaths, the continued provision of care for severely ill paediatric patients at the HDU, and the few events during these short strikes could have led us to underestimate the effect of the strikes.
