# Supplementary material for: Effect of strikes by health workers on mortality between 2010 and 2016 in Kilifi, Kenya: a population-based cohort analysis
Source: Lancet Glob Health. 2019 May 22;7(7):e961–7. doi: 10.1016/S2214-109X(19)30188-3 (PMC6560003; doi:10.1016/S2214-109X(19)30188-3)
Supplement: Supplementary appendix [file mmc1.pdf]

THE LANCET
Global Health

Supplementary appendix

This appendix formed part of the original submission and has been peer reviewed. We post it as supplied by the authors.

Supplement to: Ong'ayo G, Ooko M, Wang'ondu R, et al. Effect of strikes by health workers on mortality between 2010 and 2016 in Kilifi, Kenya: a population-based cohort analysis. *Lancet Glob Health* 2019; published online May 22. http://dx.doi.org/10.1016/S2214-109X(19)30188-3.

Supplementary table 1: **Categorisation of verbal autopsy-generated causes of death from people within the KHDSS between January, 2010, and November, 2016**

	**Deaths (%)**
**Causes of death**	
Total	5057 (100%)
**Medical cause of death (n=3656 [72%])**	
HIV/AIDS-related death	625 (12%)
Acute respiratory infect including pneumonia	575 (11%)
Stroke	417 (8%)
Other and unspecified cardiac disease	302 (6%)
Neonatal pneumonia	223 (4%)
Malaria	206 (4%)
Pulmonary tuberculosis	196 (4%)
Birth asphyxia	141 (3%)
Meningitis and encephalitis	113 (2%)
Severe malnutrition	96 (2%)
Renal failure	92 (2%)
Chronic obstructive pulmonary disease	69 (1%)
Asthma	66 (1%)
Liver cirrhosis	61 (1%)
Diabetes mellitus	59 (1%)
Diarrhoeal diseases	56 (1%)
Epilepsy	53 (1%)
Severe anaemia	52 (1%)
Other and unspecified infect disease	34 (1%)
Macerated stillbirth	33 (1%)
Neonatal sepsis	33 (1%)
Prematurity	33 (1%)
Acute cardiac disease	32 (1%)
Other and unspecified neonatal cause of death	31 (1%)
Sickle cell with crisis	17 (<1%)
Fresh stillbirth	16 (<1%)
Sepsis (non-obstetric)	12 (<1%)
Congenital malformation	8 (<1%)

Measles	3 (<1%)
Haemorrhagic fever	1 (<1%)
Pertussis	1 (<1%)
**Maternal cause of death (n=74 [1%])**	
Obstetric haemorrhage	32 (1%)
Pregnancy-related sepsis	13 (<1%)
Pregnancy-induced hypertension	12 (<1%)
Abortion-related death	6 (<1%)
Anaemia of pregnancy	6 (<1%)
Ectopic pregnancy	3 (<1%)
Other and unspecified maternal cause of death	2 (<1%)
**Medical or surgical cause of death (n=559 [11%])**	
Digestive neoplasms	193 (4%)
Other and unspecified neoplasms	148 (3%)
Respiratory neoplasms	128 (3%)
Reproductive neoplasms MF	84 (2%)
Oral neoplasms	6 (<1%)
**Other cause of death (n=50 [1%])**	
Other and unspecified non-communicable disease	41 (1%)
Other and unspecified external cause of death	9 (<1%)
**Surgical cause of death (n=212 [4%])**	
Acute abdomen	196 (4%)
Breast neoplasms	16 (<1%)
**Trauma-associated cause of death (n=506 [10%])**	
Assault	184 (4%)
Road traffic accident	127 (3%)
Accidental fall	71 (1%)
Intentional self-harm	57 (1%)
Accidental drowning and submersion	33 (1%)
Accidental exposure to smoke fire and flame	16 (<1%)
Contact with venomous plant or animal	7 (<1%)
Exposure to force of nature	6 (<1%)
Other transport accident	5 (<1%)

KHDSS=Kilifi Health and Demographic Surveillance System.

Supplementary table 2: **Effect of day of the week and public holidays on mortality in the KHDSS population between January, 2010, and November, 2016**

	**Deaths (n)**	**Person days**	**Mortality rate (per 100 000 person-days)**	**Rate ratio (95% CI)***	**p value**
**Effect of day of the week**					
Sunday	819	95 470 448	0·86	1·00	
Monday	965	95 490 560	1·01	1·18 (1·06–1·31)	0·003
Tuesday	919	95 207 136	0·97	1·13 (1·02–1·25)	0·018
Wednesday	966	95 772 216	1·01	1·18 (1·07–1·30)	0·001
Thursday	929	95 497 368	0·97	1·14 (1·02–1·26)	0·019
Friday	958	95 198 344	1·01	1·18 (1·06–1·31)	0·002
Saturday	840	95 745 640	0·88	1·02 (0·92–1·14)	0·66
Weekday	4737	477 165 632	0·99	1·00	
Weekend	1659	191 216 080	0·87	0·87 (0·82–0·93)	<0·001
**Effect of public holidays**					
Normal day	6023	632 871 488	0·95	1·00	
Public holiday	373	35 510 220	1·05	1·14 (0·99–1·30)	0·067

KHDSS=Kilifi Health and Demographic Surveillance System. *Rate ratio is adjusted for trend and seasonality in the analysis of effect of day of the week, and adjusted for trend, seasonality, and day of the week for analysis of effect of public holidays.

Supplementary Table 3: **Effect of strikes by health workers on mortality in the KHDSS between January, 2010, and November, 2016, comparing analysis excluding deaths of uncertain dates with analysis including all deaths**

	**Excluding deaths with uncertain dates**				**Including all deaths, uncertain dates set to day 15 of month**				**Including all deaths with imputation of uncertain dates of death***	
	Deaths in strike periods (n)	Deaths in non-strike periods (n)	Rate ratio (95% CI)†	p value	Deaths in strike periods (n)	Deaths in non-strike periods (n)	Rate ratio (95% CI)‡	p value	Rate ratio (95% CI)†	p value
Overall	296	6100	0·93 (0·81–1·08	0·34	459	8700	0·94 (0·83–1·08)	0·39	1·01 (0·86–1·16)	0·60
Age <1 month	32	663	0·97 (0·69–1·37)	0·88	36	720	1·02 (0·73–1·41)	0·92	1·04 (0·97–1·12)	0·81
Age 1–11 months	11	320	0·58 (0·33–1·03)	0·064	22	504	0·67 (0·43–1·04)	0·076	0·86 (0·62–1·11)	0·60
Age 12–59 months	32	356	1·75 (1·11–2·76)	0·016	44	567	1·75 (1·16–2·62)	0·0070	1·65 (1·29–2·01)	0·051
Age 5–14 years	15	330	0·90 (0·46–1·76)	0·75	18	458	0·80 (0·42–1·52)	0·50	0·98 (0·64–1·31)	0·66
Age 15–49 years	76	1391	1·01 (0·78–1·30)	0·96	123	2,023	1·02 (0·81–1·28)	0·87	1·15 (0·84–1·47)	0·48
Age ≥50 years	130	3040	0·84 (0·68–1·04)	0·10	216	4,428	0·87 (0·72–1·04)	0·13	0·90 (0·66–1·14)	0·52

KHDSS=Kilifi Health and Demographic Surveillance System. *Random imputation was done so that deaths of uncertain date were randomly assigned to any day within the month that they occurred. †Rate ratio adjusted for trend, seasonality, day of the week, and public holiday. ‡Rate ratio adjusted for trend, seasonality, day of the week, 15th day of the month, and public holiday.

Regression equation for the post-strike period analysis

$Log\lambda 1 = \alpha + \sum \beta 1,2..24X1,2\ldots 24$

And

$Log\lambda 2 = \alpha + \sum \beta 1,2..24X1,2\ldots 24$

where:

$\lambda 1$ is the mortality rate in the 1st post-strike week

$\lambda 2$ is the mortality rate in the 2nd post-strike week

α is the log(baseline mortality rate in the non-strike period)

X1 to X11 are months February to December (January is the reference month)

X12 to X17 are years 2011 to 2016 (2010 is the reference year)

X18 refers to public holidays

X19 to X24 refer to days of the week, Monday to Saturday (Sunday is the reference day)
